# Renal accumulation of prooxidant mineral elements and CKD in domestic cats

**DOI:** 10.1038/s41598-020-59876-6

**Published:** 2020-02-21

**Authors:** R. Alborough, L. Grau-Roma, S. de Brot, G. Hantke, S. Vazquez, D. S. Gardner

**Affiliations:** 1 School of Veterinary Medicine and Science, Sutton Bonington Campus, University of Nottingham, Loughborough, LE125RD United Kingdom; 2 School of Biosciences, Sutton Bonington Campus, University of Nottingham, Loughborough, LE12 5RD United Kingdom; 3 National Museums Scotland, Edinburgh, Scotland; 4 Institute of Animal Pathology, University of Bern, Bern, Switzerland

**Keywords:** Animal physiology, Kidney diseases, Kidney, Element cycles

## Abstract

Felids have a high incidence of chronic kidney disease (CKD), for which the most common renal lesion is chronic interstitial nephritis (CIN). CIN can be induced by tissue oxidative stress, which is determined by the cellular balance of pro- and anti-oxidant metabolites. Fish-flavoured foods are more often fed to cats than dogs, and such foods tend to have higher arsenic content. Arsenic is a pro-oxidant metallic element. We propose that renal accumulation of pro-oxidant elements such as arsenic and depletion of anti-oxidant elements such as zinc, underpin the high incidence of CIN in domestic cats. Total arsenic and other redox-reactive metal elements were measured in kidneys (after acid-digestion) and urine (both by inductively-coupled plasma-mass spectrometry) of domestic cats (kidneys, n = 56; urine, n = 21), domestic dogs (kidneys, n = 54; urine, n = 28) and non-domesticated Scottish Wildcats (kidneys, n = 17). Renal lesions were graded by severity of CIN. In our randomly sampled population, CIN was more prevalent in domestic cat versus domestic dog (51%, n = 32 of 62 cats; 15%, 11 of 70 dogs were positive for CIN, respectively). CIN was absent from all Scottish wildcats. Tissue and urinary (corrected for creatinine) arsenic content was higher in domestic cats, relative to domestic dogs and wildcats. Urine arsenic was higher in domestic cats and dogs with CIN. Arsenobetaine, an organic and relatively harmless species of arsenic, was the primary form of arsenic found in pet foods. In summary, the kidneys of domestic cats appear to have greater levels of pro-oxidant trace elements, as compared to dogs and wildcats. Since there was no difference in renal arsenic levels in cats with or without CIN, renal arsenic accumulation does not appear a primary driver of excess CIN in cats. Given clear differences in renal handling of pro vs. anti-oxidant minerals between cats and dogs, further *in vivo* balance studies are warranted. These may then inform species-specific guidelines for trace element incorporation into commercial diets.

## Introduction

Chronic kidney disease (CKD) is commonly diagnosed in domestic cats, with a reported prevalence of 1–3% in the overall domestic cat population^1^. One study reported prevalence as high as 50% and 80% in cats of all ages and aged over 15 years, respectively^2^. This contrasts to a reported prevalence of <1% in all dogs^3^ and only 10% in dogs over 15 years of age^1^. CKD is defined as the irreversible loss of structure and/or function of one or both kidneys that results from a progressive process occurring over a period of 3 months or more^1^. CKD has various aetiologies, but mononuclear cell tubulointerstitial nephritis – inflammation of the interstitial cells surrounding tubules, predominantly proximal – together with interstitial fibrosis and tubular atrophy is the common histopathological finding in most cases of CKD in cats^4,5^ and dogs^6^.

Renal tubular epithelial cells are highly metabolically active with high mitochondrial density in order to support oxidative phosphorylation and energy-dependent, resorptive functions^7^. Inevitably in such tissues, particularly the kidney with an end-arterial blood supply, temporary reduction of blood flow – ischaemia – can lead to increased production of reactive oxygen species (ROS^8,9^). Excess ROS can damage DNA, cause lipid peroxidation, alter intracellular protein function and increase ‘cell stress’^10^. Physiological buffering of cellular ROS is commonly by metalloenzymes such as superoxide dismutase (with Cu, Zn or Mn at the hydrolytic core), catalase and peroxidase (core of Fe), glutathione (core of Se) plus dietary anti-oxidants such as vitamins C and E^11^. Consequently, the kidney, along with the liver, is rich in anti-oxidant trace elements^11–13^. However, under some circumstances where ROS generation exceeds the reducing capacity of the resident antioxidant defences, as may often occur in the ischaemia-susceptible kidney, then structural and functional impairment including apoptosis and necrosis can occur^14^. Even single episodes of acute kidney injury can increase the risk of developing chronic kidney disease in cats^9^. Cats with CKD have higher ROS-induced renal lesions^8^. Finally, and importantly, ROS may induce inflammatory processes that underpin the development of tubulointerstitial nephritis and renal fibrosis^15^. A kidney with depleted anti-oxidant trace elements has a propensity to increased ROS generation and chronic kidney disease.

Several ‘environmentally toxic’ trace elements are pro-oxidant and actively deplete cellular anti-oxidant resources such as arsenic, cadmium, lead and molybdenum^16–18^. The kidney and liver are the two organs where these elements, particularly cadmium and arsenic, are most concentrated^19,20^. Cadmium and, particularly, inorganic arsenic can be nephrotoxic in excess^21,22^. The mechanisms in kidneys by which heavy metal exposure damage cells is multi-factorial, with increased oxidative stress and ROS production, particularly in proximal tubule epithelial cells, a contributory factor. The usual route of exposure to such elements is environmental, either through drinking water^23^ or consumption of feeds with higher content such as fish^24^, rice or wheat^25^. Since the kidneys are the primary means for excretion of water-soluble waste metabolites and toxins, urinary concentrations of many of these toxic, heavy metal elements have been used to report excess exposure^26^. In humans, high urinary total arsenic has been associated with increased risk of renal dysfunction^27^ and cadmium is a known nephrotoxin^28,29^.

For companion animals, particularly cats that have rates of CKD that far exceed other mammals, the elemental profile of kidneys in relation to kidney disease has not been studied. Indeed, we have previously described how complete pet foods with a high fish-content have increased total arsenic content^30^. Cats, more so than dogs, are often fed fish-flavoured foods^31^, despite originating from the savannah, where fish as part of a normal diet would be a rarity^32^. Chronic interstitial nephritis is the main renal lesion observed in cats with CKD and can be induced by oxidative stress. Cellular redox-balance is determined by many factors including the relative proportions of pro- and anti-oxidant mineral elements. Arsenic is one such pro-oxidant, metallic element often found at high concentration in fish-flavoured foods which are more often fed to cats than dogs. We propose that renal accumulation of pro-oxidant elements such as arsenic and depletion of anti-oxidant elements such as zinc, underpin the high incidence of CIN in domestic cats. We describe for the first time that domestic cat kidneys (cf. canine kidneys) have an elemental profile considered to be ‘pro-oxidant’; that is, relative bioaccumulation of pro-oxidant elements coupled with lower levels of elements that support anti-oxidant functions. For one such pro-oxidant element – arsenic – we further characterised the specific species (e.g. organic vs inorganic) found in four cat foods, selected as having a relatively high arsenic content. We propose that in the long-term, such an elemental microenvironment in the feline kidney may underpin the domestic cat’s propensity to CKD.

## Materials and Methods

### Ethics

This study was approved by the University of Nottingham School of Veterinary Medicine and Science Research Ethics Committee (REC 2074 170721). Consent from pet owners to use tissue from their euthanised or deceased animals for research was taken at source by a veterinarian not associated with this project. Tissues and urine from the bladder were obtained at necropsy by board-certified veterinary pathologists (LGR, SDB). All methods described were performed in compliance with the relevant guidelines and regulations for good laboratory practice.

### Collection of kidneys and urine samples

Kidneys were obtained from recently deceased or euthanised domestic cats (n = 62) and dogs (n = 70) submitted to The University of Nottingham Veterinary Pathology Service for necropsy over a 1.5-2 year period. All tissues were fixed for histopathology assessment (see below). A proportion of these kidneys (cats, n = 56; dogs, n = 54) were frozen for later analysis of tissue trace elements (see below). If present in the urinary bladder, urine (1–2 ml) was also obtained (cats, n = 27; dogs, n = 36). In addition, kidneys from a small population of recently deceased Scottish Wildcats (n = 17) were obtained from National Museums Scotland (courtesy of GH).

### Semi-quantitative scoring of chronic interstitial nephritis and fibrosis

**Figure 1:**
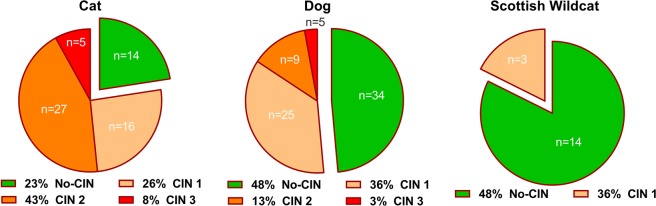
Domestic cats have higher incidence of CIN that is of a greater severity than domestic dog or Scottish Wildcat. Haematoxylin and eosin (HE) stained kidney sections from dogs, domestic cats and Scottish wildcats were scored semi-quantitatively for percentage of each field-of-view with chronic interstitial nephritis (CIN), as determined by positivity for mononuclear leukocyte infiltration. Scores of 0, 1, 2 and 3 were given where 0%, <5%, 5–25% and >26% of the FOV (n = 10/section), respectively were lymphocytic.

At necropsy, a representative block of kidney tissue, containing both cortex and medulla, was fixed in 10% neutral-buffered formalin and paraffin embedded. Formalin-fixed paraffin-embedded (FFPE) tissue was cut at 5 µm using a microtome and sections were mounted onto glass poly-L-lysine slides before staining with haematoxylin and eosin (HE). A subset were stained with Massons Trichrome (MT). All sections were mounted using DPX-mountant, according to standard protocols in our pathology department. Using light-microscopy, the severity of chronic interstitial nephritis (CIN) was estimated based mostly as the percent coverage of the tissue section affected with interstitial mononuclear inflammatory infiltrate. A semi-quantitative scoring system was developed from previously reported methods^4,5^. In brief, scores of 0 (absent), 1 (mild), 2 (moderate) and 3 (severe) were given where 0%, <5%, 5–25% and >25% of the tissue section, respectively was positive for lymphoplasmacytic inflammation (CIN) or MT-positive fibrosis (Supplementary Information, Figure S1). In addition, the degree of fibrosis was semiquantified as absent (0), mild (1), moderate (2) or severe (3) in a proportion of animals using MT. A score of 0 and 1 were designated ‘No-CIN’ whereas scores of 2 and 3 were ‘CIN’. Two researchers (LG-R, RA) independently scored each kidney tissue section and compared findings (<10% of all cases). Where different scores had independently been attributed, the case was re-evaluated and a mutual agreement on scoring and classification reached. After all slides had been evaluated, n = 11 of 70 dogs and n = 32 of 62 domestic cats showed lesions consistent with CIN scores 2 and 3 (Fig. 1). The animals’ pathology reports were obtained and descriptive data reported (age, breed, other diagnoses). Cases with a known renal infectious and/or nephrotoxic aetiology of CIN such as canine herpes virus, feline infectious peritonitis, bacterial pyelonephritis or ethylene glycol toxicity were excluded. Post-mortem reports were not available for Scottish wildcats. Available data included only the location in which they were found, the macroscopic appearance of the kidneys and the species identification as a ‘Scottish Wildcat’. Five dogs and five domestic cats were of unknown neutered status.

### Preparation of kidney, urine and pet food samples for trace element analysis

Due care was taken at all times to prevent inadvertent sample contamination. Approximately 1 g of frozen kidney tissue (predominately cortex but also part-medulla) was cut and its weight recorded in mgs then freeze-dried over a period of 48–72 hours. Dry weights were recorded to a precision of 0.10 mg and percentage water content calculated. A known amount of freeze-dried kidney tissue (min, 100 mg; max, 250 mg) was added into 50 ml inert PTFE-TFM-lined digestion vessels and acid digested using 3.0 ml of 68% trace analysis grade HNO_3_, 2.0 ml of 30% H_2_O_2_ (both Fisher Scientific UK Ltd, Loughborough, UK) and 3.0 ml deionised milli-Q water (18.2 MΩ.cm). Alongside every 20 digestion vessels containing samples (10 samples in duplicate), two tubes containing 100–200 mg of a certified reference material (CRM; NIST, National Institute of Standards and Technology, Gaithersburg, MD, USA; 1577c bovine liver), plus two ‘blank’ tubes containing only digestion chemicals, were used. The digestion process was accelerated using the Multiwave PRO microwave reaction system and the 24HVT50 rotor (Anton Parr, Austria). Over a total of 45 minutes, samples were heated to 140°C over 10 minutes, held at this temperature for 20 minutes and then cooled to 55°C for the remainder. After acid-digestion, the tube was further diluted to a total volume of 15.0 ml by addition of 7.0 ml of deionised milli-Q water. From this tube 500 µl was used for trace element analysis by Inductively-coupled plasma mass spectrometry (ICP-MS; iCAPTM Q, Thermo-Fisher Scientific Inc., Waltham, MA, USA). Four freeze-dried and homogenised cat foods with high arsenic content, as determined previously^30^, were selected and underwent the same acid digestion process prior to total arsenic measurement by ICP-MS. For pet foods, triplicate samples were prepared and run alongside three ‘blanks’ and 100–200 mg of fish muscle ERM-BB422 CRM, also in triplicate. Total arsenic was also measured in these four pet foods, fish muscle CRM and ‘blanks’, all in triplicate, after water-extraction as described below. For urine, samples were thawed at room temperature, centrifuged for 5 minutes (800 *g*) and a 500 µl neat sample used for ICP-MS. For every ≤60 urine samples, duplicate CRM samples (SERO210705 Seronorm Trace Elements Urine L-2; LGC Standards) were used for quality control purposes.

### Urine creatinine measurement

Creatinine was measured in neat, urine samples (200–500 µl) using an automated assay, based on the enzymatic reaction of creatinine with picric acid producing a colormetric product (RX-Imola; Randox Laboratories Ltd, London, UK). The assay and standards were according to the manufacturer’s instructions, and included human serum (clinical chemistry calibration serum level 3, CAL2351) and urine (assayed urine control level 3, AU2353) with known creatinine levels as quality controls, diluted serially from 16,000 µmol/L. Before adjustment of urine elements (µg/L) to urine creatinine (µmol/L), the latter was first converted to µg/L using its molecular weight of 88.42 g/mol, and then the ratio expressed as µg element per g creatinine.

### Water-extraction of arsenic species in cat foods with high arsenic content

Four fish-containing pet foods with high arsenic, as determined previously^30^, were selected for arsenic speciation. Purite water was used to extract arsenic species according to the procedure outlined by Komorowicz, *et al*.^33^ Here, microwave-assisted extraction of arsenic species in freshwater fish with water, as opposed to a water-methanol solution, gave the best recoveries, which ranged from 92 to 99%. In brief, 8 ml of purite water was added to each 200–300 mg sample of freeze-dried, homogenised pet foods and a fish muscle certified reference material (CRM; ERM-BB422). Each pet food and the CRM was extracted in triplicate. The microwave (power 800 W) was ramped to 80°C over 10 minutes, held at this temperature for 15 minutes and then cooled over 10 minutes. After this, the contents of the microwave vessels were decanted into appropriately labelled centrifuge tubes and rinsed with 1 ml deionised water, which was then added to the appropriate sample. The samples were centrifuged at 2000 g for 10 minutes and the supernatant was transferred to universal tubes using a Pasteur pipette. Care was taken to avoid collecting any of the undissolved sediment.

### High performance liquid chromatography-coupled inductively coupled plasma-mass spectrometry conditions for arsenic speciation

0.6 ml of each sample was transferred into plastic HPLC PolyVials with filter caps (Thermo-Fisher Scientific Inc., Waltham, MA, USA). Arsenic speciation was undertaken using high performance liquid chromatography coupled to inductively coupled plasma mass spectrometry (HPLC-ICP-MS; HPLC 5000 series) with a PRP-X100 anion exchange column (PS-DVB/Trimethyl ammonium exchanger; 5 µm particle size; 4.6 mm internal diameter, ID; 250 mm length) in a temperature controlled compartment at 40 °C. The eluent was 20 mM NH_4_H_2_PO_4_ and (NH_4_)_2_HPO_4_ at pH = 5.6, pumped at 1.5 ml/minute in isocratic mode. Standards included 5.0 µg/L arsenite (As[III]) and arsenate (As[V]) (Spex Certiprep, Stanmore, U.K.), and 5.0 µg/L dimethylarsinic acid (DMA), monomethylarsinic acid (MMA) and arsenobetaine (AsB) (Sigma/Merck, Darmstadt, Germany). Each pet food sample and the fish muscle CRM was also spiked with 5.0 µg/L of the AsB standard to separate overlapping peaks of AsB and As(III) in the samples (e.g. see Figure S2, Supplementary information). Chromatography run-time was around 13 minutes per sample. The output of the ICP detector, an electron multiplier detector, was the number of pulse counts (ions arriving) per second (counts per second, cps). Interpolation between calibration standards, which were run periodically, gave individual sensitivity values, in cps *per* parts per billion (cps/ppb), for each sample.

### Mass-spectrometry conditions for measurement of trace elements

Conditions for ICP-MS were as described previously^30^. Briefly, 500 µl of water-extracted and acid-digested samples were diluted by the addition of 9500 µl of double line 0.5% HNO_3_ into 14 ml (105 mm × 16.8 mm) Polypropylene tubes (Sarstedt, Leicester UK). ICP-MS (Thermo-Fisher Scientific Inc., Waltham, MA, USA) used a He collision cell with ‘kinetic energy discrimination’ to reduce polyatomic interference. 30 elements can be determined (Ag, Al, As, Be, B, Ba, Cd, Ca, Co, Cr, Cs, Cu, Fe, K, Li, Mg, Mn, Mo, Na, Ni, P, Pb, Rb, S, Ti, Sr, Tl, U, V and Zn). Li, Be and P are determined in standard (vacuum) mode, Se in ‘hydrogen-cell’ mode. In-sample switching allows for simultaneous measurement. Sc, Ge, Rh and Ir are used as internal standards. Tissue elemental composition is presented after correction for blanks and batch variation (using the CRM as reference) as ppm (e.g. major elements, 1 ppm = 1 mg/kg) or ppb (e.g. trace elements, 1 ppb = 1 µg/kg) or per 100 g of dry matter [DM] as indicated in the text. For major and trace elements, recovery was > 95% with < 10% coefficient of variation for each (n = 24 separate analyses). Intra-assay variability for all elements presented in this manuscript was <2%. Variability due to elements often being below limits of detection (LOD; µg/kg) in many kidneys was relatively high for titanium, lithium, beryllium, silver and aluminium. These elements are therefore not reported. Elements near to or below LOD are also not reported (vanadium, median in kidney tissue = 0.085 (LOD = 0.163); chromium, 0.156 (0.488); cobalt, 0.051 (0.064); nickel, 0.110 (0.633); caesium, 0.052 (0.072); barium, 0.128 (0.647); thallium, 0.004 (0.059); lead, 0.065 (0.211); uranium, 0.003 (0.017). Limits of detection were determined from the standard deviation of n = 10 blanks.

### Statistical analysis

Data were analysed using analysis of variance (ANOVA) for the fixed effects of species (dog, cat, wildcat) and CIN. Scores of 0 and1 corresponded to animals with very few mononuclear infiltrates within the renal interstitium (less than 5%), and therefore very little pathological significance versus scores of 2 and 3 with considerable inflammatory infiltrate and a high likelihood of pathological significance. Pre-specified interactions (species ± CIN) were also considered. For some analyses, the design was unbalanced, since none of the Scottish Wildcats had CIN (when CIN scores 0 and 1 were grouped together as ‘No CIN’). In this instance, to compare domestic cat vs wildcat, the Mann-Whitney U-test was used. All data were analysed using GenStat v19 (VSNi Ltd., Rothamsted, UK). In order to meet assumptions for analysis by ANOVA all data were first checked for a normal distribution of residuals. If necessary, data were log-transformed (log_10_) to normalise the distribution of the data prior to analysis by ANOVA. For the small number of cases where either sex of the animal or neutered status was unknown, but experimental data were available, then a different approach was taken so as not to exclude that individual from all analyses. These data were analysed by linear mixed model, restricted maximum likelihood (REML) as missing values are assumed to be at random and therefore do not bias the estimates. Finally, multivariate linear discriminant analyses were used as an objective means to effectively demonstrate patterns in complex (i.e. multiple variates), potentially non-independent data using orthogonal partial least squares discriminant analysis (OPLS-DA; SIMCA-P v15, Umetrics, Umea, Sweden).

### Power and sample size

Sample size for the study was estimated from a pilot study of 35 kidney tissue samples comprising n = 15 dogs and n = 20 cats in which measured kidney tissue arsenic concentration (µg/g DM^−1^) in cats without CKD (No-CIN) was 0.164 ± 0.037 versus cats with CIN (0.612 ± 0.350). Using a replication ratio of 1.0 and treatment mean effect size, standard deviation and variance of 0.550, 1.10 and 1.21 µg/g DM^−1^, respectively, we estimate we would need at least 51 individuals per treatment group (dogs vs cats) to be able to reject the null hypothesis that the population means are equal with probability (power) 0.80, if the true difference in the sample means of control vs. CIN kidney was > 0.550 µg/g DM^−1^. The Type I error probability associated with this test of the null hypothesis is 0.05.

## Results

### Descriptive statistics and estimation of chronic interstitial nephritis and fibrosis

From our sample collected over an 1.5-2 year period from the UoN-VPS, based on the interstitial inflammation, the incidence of CIN *per se* was greater in domestic cats (77% [48 of 62 examined]) versus dogs (51% [36/70]; P < 0.01 by χ^2^ test). Furthermore, domestic cats had a greater incidence of moderate-to-severe CIN (51% [32/62]) than dogs (15% [11/70]; P < 0.001 by χ^2^ test) (Fig. 1). The majority of Scottish Wildcats showed no inflammatory cells within the interstitium (score = 0), but three of 17 (18%) showed few multifocal and small aggregates of mononuclear cells within the interstitium (score = 1). In a randomly selected proportion of kidneys from cats and dogs with or without CIN, renal fibrosis was assessed using Massons Trichrome (MT) staining and scored accordingly. All (100%) domestic cats (10/10) and 80% dogs (12/15) showed an increased amount of interstitial collagenous tissue (fibrosis) which was evident by using MT. Two of 7 wildcats showed a mild increase in the amount of interstitial collagenous tissue but both lacked the presence of any inflammatory infiltrate within the interstitium. Similar to the amount of interstitial inflammation, moderate-to-severe fibrosis was identified in a greater proportion of domestic cat than dog kidneys (Cat, 90%; 9/10; Dog, 40%; 6/15). There was good concordance in kidneys scored for both mononuclear interstitial inflammation and fibrosis (0.64; where <0.25 = no agreement and >0.75 = strong agreement; Linns coefficient of concordance). Retrospective analysis of our database of submitted tissue indicated that the average age (in months) of domestic cats and dogs with CIN was much older, as expected (Cats; No-CIN, 58 [17–96] vs. CIN, 166 [113–207] months: Dogs, No-CIN, 44 [11–90] vs. CIN, 78 [29–150]; median [IQR], P < 0.001). The proportion of males within each category was not different in cats (No-CIN, 21 of 37 (56%) vs. CIN, 20 of 37 (54%) but was higher in dogs with CIN (No-CIN, 20 of 46 (43%) vs. CIN, 10 of 12 (83%); χ^2^ value = 6.54, 1_*df*_, P = 0.02). The majority of cats (84%) but only half the dogs (51%) had been neutered.

### Elemental composition of kidneys

**Figure 2:**
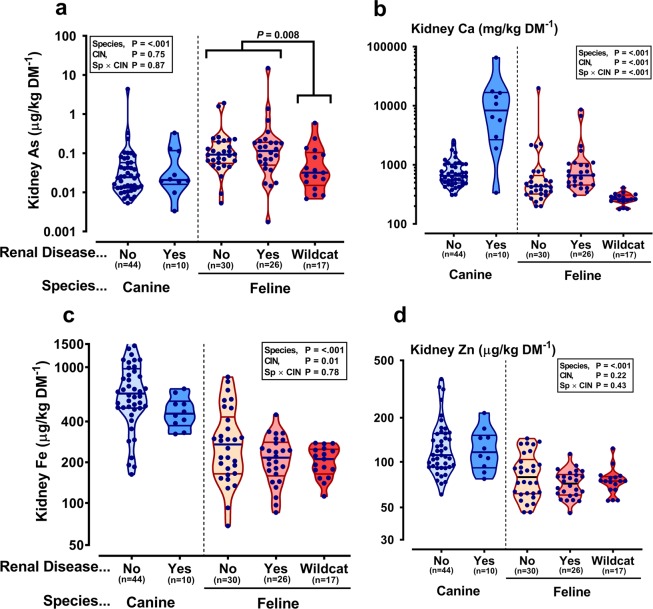
Domestic cats have higher kidney arsenic content than domestic dog or wildcat. Data are kidney tissue arsenic (As; **a**), calcium (Ca; **b**), iron (Fe; **c**) and zinc (Zn; **d**) content in dogs and cats with (Renal Disease: Yes) or without (Renal Disease: No) chronic interstitial nephritis (CIN), and in Scottish Wildcats – all without CIN. Violin plots represent all data-points with middle line at median flanked by lower and upper quartiles. Elements were determined by ICP-MS after freeze-drying and acid-digesting samples. The y-axis is on a logarithmic scale. Data were log_10_-transformed prior to statistical analysis, which was by linear mixed models, restricted maximum likelihood (REML) with species and CIN as fixed effects, accounting for variable proportions of males and females in each group and the number that were neutered or not. Statistical significance was accepted at P = 0.003 to account for multiple comparisons. For kidney arsenic, the statistical significance of the difference between domestic and wildcat kidneys was analysed by the Mann-Whitney U test.

Total arsenic, whilst at relatively low levels in the majority of kidneys, was significantly higher in domestic cat than dog kidneys *per se*, regardless of a histopathological diagnosis of CIN (Fig. 2a). Interestingly, Scottish wildcats had significantly lower total arsenic relative to domestic cats (Fig. 2a). Both dogs and domestic cats with CIN had significantly increased kidney calcium content, which was exacerbated in dogs (Fig. 2b). Magnesium, phosphorus, potassium and sodium (of the major elements; Table 1) and iron (Fig. 2c), zinc (Fig. 2d) copper, molybdenum, rubidium and strontium (of the trace elements; Table 1) were all significantly lower in domestic cat versus dog kidneys. Only phosphorus and rubidium were also different in domestic cat vs. Scottish wildcat, each being significantly lower in the domestic cat. CIN appeared to have little overall effect on the elemental composition of kidneys, with the exception of copper, which was reduced in CIN kidneys, and strontium, which was elevated by CIN in kidneys from both cats and dogs (Table 1). Interestingly, phosphorus was significantly elevated in CIN dog kidneys, but reduced in CIN cat kidneys (Table 1).

**Table 1:** Major and trace elements in kidneys of domestic dogs and cats with or without CIN and in Scottish Wildcats.

Major element (mg/kg DM^−1^)	Dogs (n = 54)		Cats (n = 56)		Wildcats (n = 17)	Statistics, *P*-value		
	No-CIN (n = 44)	CIN (n = 10)	No-CIN (n =30 )	CIN (n = 26)	No CIN (n = 17)	Species	CIN	Sp x CIN
Magnesium	681 (640, 740)	788 (705, 1199)	558 (463, 685)	537 (476, 632)	506 (467, 595)	** < 0.001**	0.27	0.004
Phosphorus	10492 (9665, 11268)	11776 (10944, 17246)	8865 (7372, 10590)	8799 (7481, 9931)	9634* (9181, 11127)	< 0.001	0.008	** < 0.001**
Potassium	9470 (8645, 10928)	9596 (8551, 11125)	8343 (7035, 9917)	8149 (6639, 9274)	8043 (7487, 8522)	**0.003**	0.87	0.052
Sodium	11609 (10184, 13663)	12637 (10389, 14579)	10133 (7702, 11181)	9766 (8221, 11949)	6143 (5236, 7611)	** < 0.001**	0.11	0.27
Sulphur	9763 (8802, 11216)	9947 (8741, 10897)	8488 (7073, 9575)	8730 (7919, 9779)	7227 (6857, 7487)	0.05	0.35	0.70
**Trace element (µg/kg DM**^**−1**^**)**								
Cadmium	1.29 (0.62, 1.69)	0.58 (0.29, 1.57)	0.30 (0.16, 0.59)	1.02 (0.78, 1.69)	0.25 (0.15, 0.40)	0.029	0.009	0.010
Copper	44.6 (29.1, 64.4)	12.5 (10.3, 27.4)	10.2 (8.2, 14.1)	9.65 (8.3, 11.4)	11.3 (9.5, 11.8)	** < 0.001**	** < 0.001**	0.012
Manganese	4.74 (3.98, 6.36)	3.06 (2.11, 3.80)	3.88 (3.15, 4.79)	4.04 (3.38, 4.39)	3.69 (2.89, 4.19)	0.32	0.015	0.015
Molybdenum	1.01 (0.77, 1.33)	0.73 (0.51, 1.10)	0.46 (0.35, 0.71)	0.46 (0.33, 0.56)	0.64 (0.56, 0.74)	** < 0.001**	0.10	0.886
Rubidium	19.6 (15.0, 23.6)	17.0 (15.3, 19.8)	11.6 (8.46, 15.7)	11.1 (8.41, 13.0)	23.6* (17.4, 48.1)	** < 0.001**	0.28	0.34
Selenium	4.55 (3.87, 5.55)	3.87 (3.17, 5.61)	4.45 (3.52, 5.01)	4.15 (3.12, 5.11)	4.31 (3.74, 5.79)	0.048	0.15	0.58
Strontium	0.41 (0.31, 0.71)	2.73 (1.27, 8.5)	0.27 (0.20, 0.39)	0.57 (0.28, 1.15)	0.12 (0.09, 0.17)	** < 0.001**	** < 0.001**	0.015

Data are presented as median (IQR; 1st to 3rd quartile) and are representative of duplicate samples analysed by ICP-MS. Statistical comparisons were made between domestic dogs and cats with or without CIN as a 2 (cat vs dog) × 2 (No-CIN vs CIN) including the prespecified interaction by ANOVA. Batch ID was included as a random effect. Variability associated with sex and neuter status were included in the statistical model, but are not reported independently as these were not primary outcomes of the analysis. Copper, cadmium and strontium were log10 transformed prior to analysis to normalize residual errors, avoiding distributional bias. Comparison between domestic and wildcats (No-CIN only) was conducted separately using similar models as previously described. Statistical significance was accepted at P < 0.003 (Bonferroni correction for 14 elements above LOQ). *indicates difference between domestic and wildcat at P < 0.003.

### Elemental composition of urine (corrected to creatinine)

**Figure 3:**
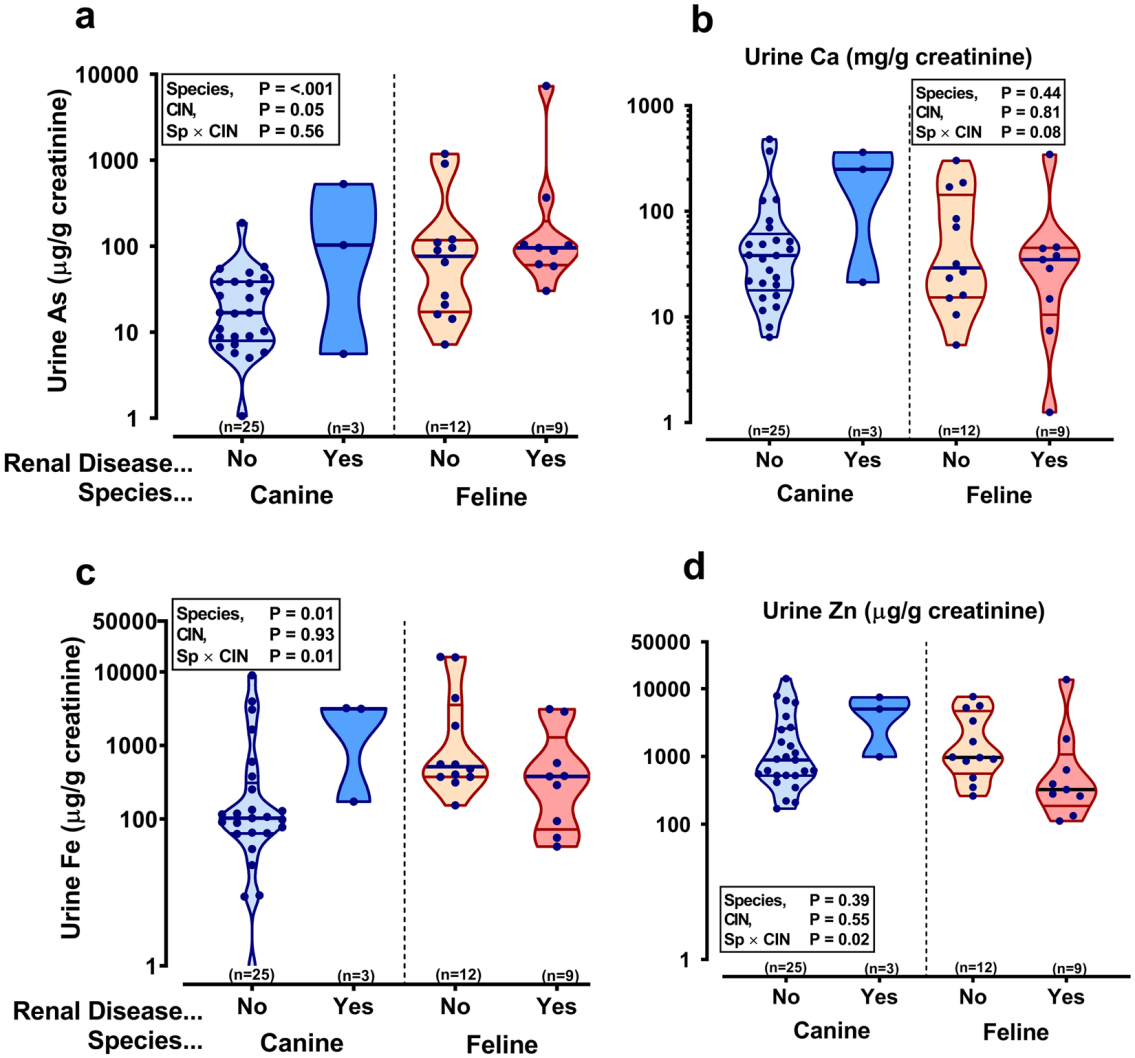
Domestic cats have lower urine calcium and zinc than domestic dog. Data are urine arsenic (As; **a**), calcium (Ca; **b**), iron (Fe; **c**) and zinc (Zn; **d**) corrected on a mass-specific basis to urine creatinine (µmol/L × 88.4) for spot-sampling of the bladder at post-mortem in dogs and cats with (Renal Disease: Yes) or without (Renal Disease: No). Renal disease was classified by presence of chronic interstitial nephritis (CIN). Violin plots represent all data-points with middle line at median flanked by lower and upper quartiles. Elements were determined by ICP-MS. The y-axis is on a logarithmic scale. Data were log_10_-transformed prior to statistical analysis, which was by linear mixed models, restricted maximum likelihood (REML) with species and CIN as fixed effects, accounting for variable proportions of males and females in each group and the number that were neutered or not. Statistical significance was accepted at P = 0.003 to account for multiple comparisons.

**Figure 4:**
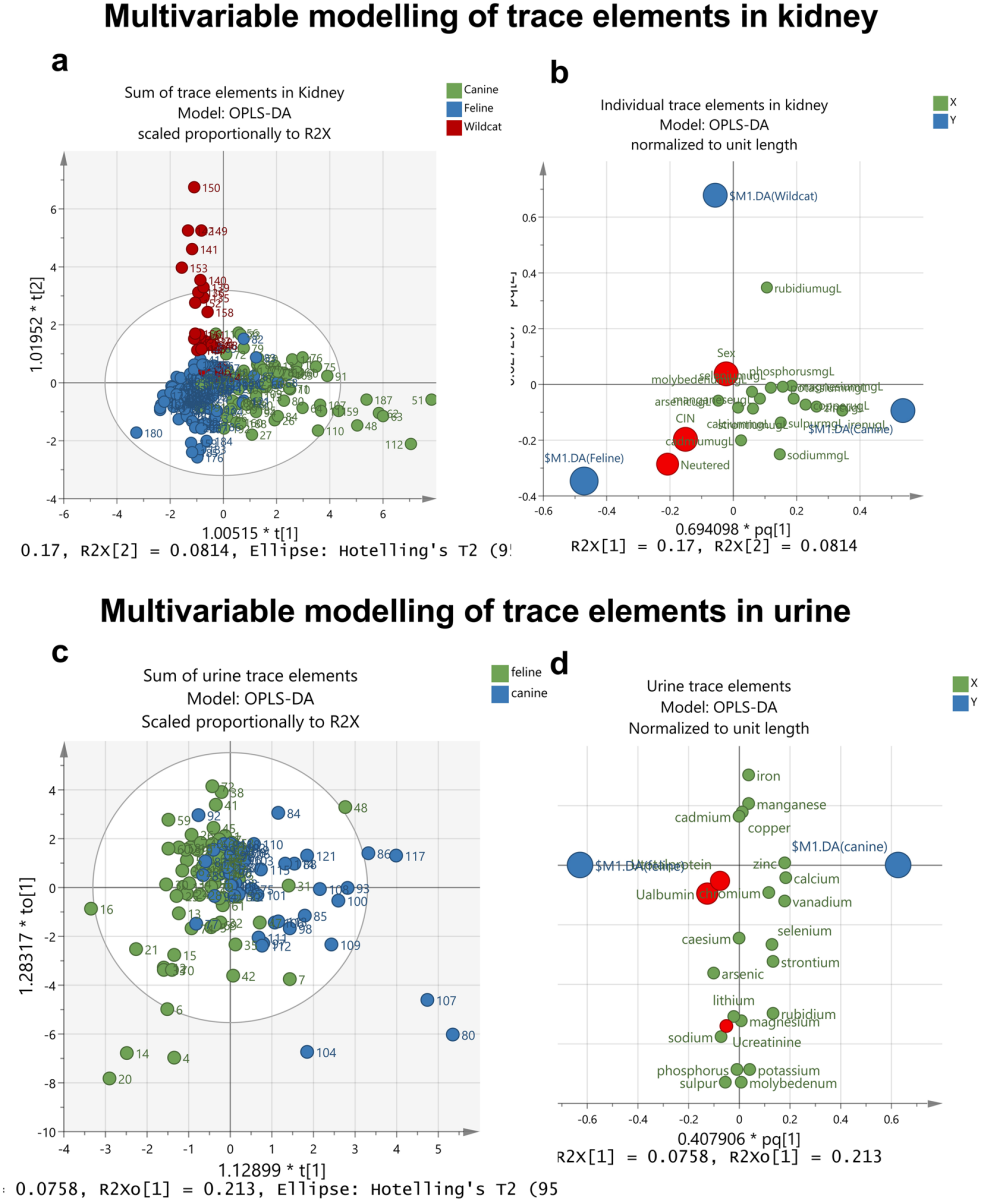
Urinary trace element profile is similar in cats and dogs. Multivariate analysis incorporating all urine samples collected, including those of unknown sex or neutered status, was used to identify any discriminating elements according to species (canine or feline) or CIN using orthogonal partial least squares - discriminant analysis (OPLS-DA; SIMCA, Umetrics Ltd, Umea, Sweden). (**a,c**) scores attributable to individual samples from feline, canine or wildcat groups in kidney (**a**) or urine (**c**); (**b**) loadings of individual elements in the three groups in kidney (**b**) or urine (**d**). OPLS-DA indicates elements contributing toward most variability in the groups. Scores at 0 on both axes do not contribute toward any variability. Scores in direction of that group suggest those parameters define that group to a greater extent than the others; for example, CIN and neutered are more prevalent in feline whilst kidney rubidium contributes most variability in wildcat kidneys (Fig. 4b).

Urinary arsenic was higher in animals with CIN and tended toward being higher in domestic cats *per se* (Fig. 3a). Similar to kidney, urinary calcium was higher in dogs relative to domestic cats (Fig. 3b) whereas zinc was also lower in the urine of domestic cats relative to dogs (Fig. 3d). No significant differences were found for urine iron (Fig. 3c). Most other measurable elements in urine were also similar between domestic cats and dogs (Table 2), with the exception of potassium and magnesium; for both, CIN tended to increase the relative concentration in urine in dogs but either decrease or not affect levels in domestic cats (Table 2). Unbiased, multivariate discriminant analysis of all samples and of all measurable elements, including those with missing secondary information such as sex and neutered status, was able to discriminate by species, well (Fig. 4a). However, the majority of individual elements in kidney (Fig. 4b) and in urine (Fig. 4c,d) were centred, and did not contribute significantly toward any discrimination by disease status (i.e. susceptibility to CIN) in domestic feline, versus the known lower prevalence in domestic canines or wild felines.

**Table 2:** Major and trace elements in urine of domestic dogs and cats with or without CIN.

Major element (mg/g creatinine)	Dogs (n = 36)		Cats (n = 27)		Statistics, *P*-value		
	No-CIN (n =30 )	CIN (n = 6)	No-CIN (n = 14)	CIN (n = 13)	Species	CIN	Sp x CIN
Magnesium	45.6 (15.5, 86.1)	196 (63.2, 334)	44.1 (18.8, 73.9)	21.0 (15.1, 62.6)	0.015	0.003	** < 0.001**
Phosphorus	1044 (326, 1672)	1678 (1545, 3838)	888 (653, 1846)	952 (740, 1961)	0.57	0.12	0.069
Potassium	3026 (1541, 4480)	6209 (3709, 15228)	2662 (1656, 4658)	2610 (1539, 4666)	0.26	0.04	**0.006**
Sodium	1826 (681, 4363)	6629 (1445, 14688)	1857 (1130, 3123)	3475 (1117, 5221)	0.48	0.17	0.18
Sulphur	1146 (685, 1541)	2045 (1579, 4527)	1123 (880,1683)	1135 (753, 1586)	0.88	0.73	0.11
**Trace element (µg/g creatinine)**							
Cadmium	0.14 (0.08, 0.31)	0.38 (0.16, 0.94)	0.05 (0.03, 0.26)	0.07 (0.04, 0.13)	0.55	0.22	0.97
Copper	157 (58.0, 419)	424 (111, 1389)	70.9 (38.9, 171)	69.0 (33.7, 125)	0.42	0.89	0.24
Manganese	3.06 (1.54, 5.20)	4.19 (3.72, 19.8)	2.04 (0.24, 6.18)	3.12 (0.42, 6.33)	0.16	0.38	0.50
Molybdenum	20.7 (8.77, 72.6)	80.9 (13.4, 141)	27.2 (12.1, 58.1)	14.0 (5.6, 30.5)	0.22	0.43	0.04
Rubidium	2350 (1390, 4561)	3628 (3062, 8279)	1382 (867, 3079)	2196 (973, 2980)	0.02	0.27	0.20
Selenium	88.8 (50.8, 121)	106 (85.1, 254)	78.2 (55.9, 100)	65.6 (53.8, 96.7)	0.20	0.95	0.42
Strontium	105 (53.2, 172)	198 (115, 258)	39.1 (20.9, 132)	101 (29.2, 436)	0.35	0.08	0.99

Data are presented as median (IQR; 1st to 3rd quartile) and are representative of duplicate samples analysed by ICP-MS. Statistical comparisons were made between domestic dogs and cats with or without CIN as a 2 (cat vs dog) × 2 (No-CIN vs CIN) including the prespecified interaction by ANOVA. Batch ID was included as a random effect. Variability associated with sex and neuter status were included in the statistical model, but are not reported independently as these were not primary outcomes of the analysis. Copper, cadmium and strontium were log10 transformed prior to analysis to normalize residual errors, avoiding distributional bias. Comparison between domestic and wildcats (No-CIN only) was conducted separately using similar models as previously described. Statistical significance was accepted at P < 0.003 (Bonferroni correction for 14 elements above LOQ). *indicates difference between domestic and wildcat at P < 0.003.

### Total arsenic and arsenic species in fish-containing pet foods

**Figure 5:**
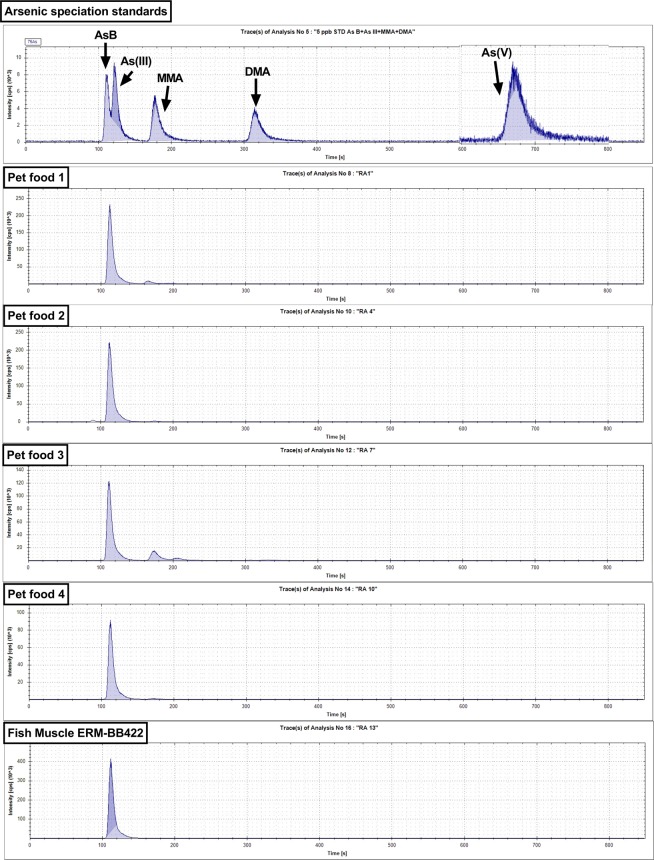
Representative chromatograms of arsenic species in wet cat food and fish muscle CRM. Representative chromatograms of arsenic species in: top) standard solution of 5.0 µg/L arsenobetaine (AsB), arsenite (As(III)), monomethylarsinic acid (MMA), dimethylarsinic acid (DMA) and arsenate (AsV), corresponding to retention times of ~110 s, 125 s, 175 s, 310 s and 660 s, respectively. Chromatogram for AsV is overlaid for clarity. For original see Supplementary information Figure S2. Middle four: corresponding to four pet foods selected as having high total arsenic content; bottom) certified reference material (fish muscle ERM-BB422), with high AsB content.

Total arsenic (TAs) concentrations measured in acid-digested and water-extracted samples were similar, but values tended to be higher in the water-extracted samples (Table 3). The mean TAs concentrations measured in acid-digested and water-extracted fish muscle ERM-BB422 CRM were compared to the certified value of TAs for the CRM (1.28 ± 0.7 mg 100 g DM) and were found to be 1.50- and 1.53-times higher, respectively. Percentage recovery ranged from 72.44 to 84.72% and from 65.59 to 74.62% in the different pet foods when the sum of arsenic species measured was calculated as a percentage of the total elemental arsenic as measured in our acid digested (‘D-TAs’; Table 3) and water-extracted samples (‘E-TAs’; Table 3), respectively. AsB was the predominant arsenic species detected in all pet food samples (Table 3; Fig. 5). Other arsenic species, such as DMA and MMA were barely detectable in any sampled food (Table 3). Inorganic and toxic arsenic species (e.g. As(V) As(III)) were not detected in any pet food or fish muscle standard (Table 3; Fig. 5 and S2, Supplementary information).

**Table 3:** Concentration of total arsenic and arsenic species in selected samples of wet cat food and Fish muscle CRM.

Sample	Mean Concentration ± Standard Deviation (mg/100 g DM)							As Recovery (%)	
	D TAs	E TAs	E AsB	E DMA	E MMA	E As(V)	ΣAs Species	D AR	E AR
Pet food 1	0.9926 ± 0.040	1.1269 ± 0.103	0.7870 ± 0.040	0.0399 ± 0.002	0.0048 ± 0.0007	0.0093 ± 0.001	0.8410 ± 0.040	84.72	74.62
Pet food 2	0.7315 ± 0.026	0.9032 ± 0.048	0.5773 ± 0.019	0.0061 ± 0.0002	0.0036 ± 0.0006	0.0054 ± 0.0006	0.5924 ± 0.020	80.98	65.59
Pet food 3	0.5524 ± 0.033	0.5510 ± 0.082	0.3215 ± 0.012	0.0696 ± 0.004	0.0069 ± 0.0007	0.0021 ± 0.0008	0.4002 ± 0.015	72.44	72.63
Pet food 4	0.3242 ± 0.004	0.3936 ± 0.014	0.2579 ± 0.024	0.0036 ± 0.0002	0 ± 0	0 ± 0	0.2615 ± 0.024	80.67	66.44
Fish muscle CRM	1.9059 ± 0.029	1.9493 ± 0.047	1.2524 ± 0.017	0.0015 ± 0.0002	0 ± 0	0 ± 0	1.2538 ± 0.017	65.79	64.32

Mean (n = 3) and standard deviation of total arsenic (TAs) and arsenic species (AsB – arsenobetaine; DMA – dimethylarsinic acid; MMA – monomethylarsinic acid; As[V] – arsenate) concentrations (mg/100 g dry matter [DM]) in selected samples (n = 4) of wet cat food with high total elemental arsenic content and fish muscle ERM-BB422 certified reference material that were either acid digested (D) or water-extracted (E). Arsenic recovery (AR) was calculated as the percentage of the total arsenic concentration that the sum of arsenic species amounted to (ΣAs Species).

## Discussion

We proposed that domestic cats, with a higher than average intake of fish-based foods compared to domestic dogs and Scottish wildcats, have a higher intake of dietary arsenic that bioaccumulates in their kidneys, leading to interstitial nephritis that underpins CKD. Our data only partially support this contention. In our sampled population of domestic cats and dogs submitted to the University of Nottingham veterinary pathology service, we found a high incidence of renal lesions in domestic cats, whom had increased levels of arsenic in their kidney tissue and urine, relative to domestic dogs and Scottish wildcats. However, total arsenic content in kidney and urine were very low *per se*. Whilst, animals with CIN also had higher urine arsenic, any differences were again biologically small. Furthermore, we determined the form of arsenic present in companion animal foods and thus likely to bioaccumulate in their tissues, to be almost entirely arsenobetaine – an organic, non-toxic arsenic species. Nevertheless, we show for the first time that a feral felid, the Scottish wildcat, had barely detectable renal lesions, despite the size of their kidneys indicating a fully-grown adult animal. In contrast, it is relatively rare for adult domestic cats to be free from any renal lesion. Thus, our data compliments other work to suggest that domestic environment appears a risk-factor *per se* for cats to develop CKD. Whilst diet and genetics may partly underpin this risk, we exclude the possibility that increased intake of organic arsenic, as is often found at high levels in fish-based diets, is a likely causative factor.

### Renal histopathology and prevalence of chronic interstitial nephritis in felids

In our population of domestic cats and dogs from a regional pathology unit over an 1.5-2 year  collection period we found, unsurprisingly, that more kidneys from domestic cats, than those from dogs and Scottish wildcats, had CIN. A high proportion of those cats had moderate-severe CIN. This corroborates prior work indicating that CKD, for which CIN is the most common renal lesion^4,34^, is frequently observed in domestic cats, but not in dogs^1^. Similar to the current study, Chakrabarti and colleagues observed a correlation between scores for renal inflammation and fibrosis^4^. However, McLeland *et al*. reported that scores for inflammation were generally greater than scores for fibrosis at each stage of CKD^5^. This suggests, as is commonly thought, that inflammation generally precedes fibrosis in any organ^35^. In the current study, analysing mineral content of kidney tissue and urine from cats and dogs, grouped by the presence or absence of mononuclear cell inflammation (CIN), as opposed to fibrosis score, will have allowed identification of a greater number of potential cases of CKD, including those at an earlier stage of progression to later CKD. Presence of inflammation in the renal interstitium has been associated with azotaemia in cats^4,5^. Thus, while clinical parameters, such as serum creatinine, used to assess the severity of azotaemia and thus stage CKD according to the IRIS system, were not measured in the current study, it is likely that our CIN scores 2 and 3 are indicative of CKD. However, direct correlation between the severities of interstitial inflammation and azotaemia is not a consistent finding^4,5^. Therefore, it should be noted that the CIN scores used in the current study indicate differing degrees of CIN and not necessarily the differing severity of CKD.

It has also been noted that when captive or truly-wild cat sub-species such as cheetahs, leopards, lynx, cougars, jaguars, ocelots, lions and tigers have been necropsied, minimal inflammatory aggregates and fibrosis are occasionally observed, similar to the data presented here. The Scottish wildcat *(Felis silvestris grampia)* is one of three main sub-species of wildcat *(Felis silvestris)*^36^. However, most domestic cats are believed to descend from another wildcat sub-species, the African wildcat (*Felis silvestris lybica)*^37^. Wildcats have not inhabited England, Wales or southern Scotland since the mid-19^th^ century; in fact, the Scottish wildcat is endemic to northern Scotland^38^. Despite probable introgression with domestic cats, creating a population of wild-living cats possessing both domestic- and wild-cat genes^39^, the Scottish wildcat can be distinguished from the domestic cat *(Felis silvestris catus)* and the European wildcat *(Felis silvestris silvestris)* based on coat colour, tail shape and markings, skull measurements and gut length^36^. The Scottish wildcat is truly wild and has been a protected species under Schedule 5 of the Wildlife and Countryside Act since 1988^39^. Thus, our finding of higher prevalence of CKD in domestic cats than in the Scottish wildcat, may be attributable to aspects of domestication such as diet fed. Nevertheless, we cannot exclude either a subtle genetic contribution, since Scottish wildcats have been proposed as a distinct species, or other environmental factors that may contribute to differences in disease prevalence between the two populations of felids. For example, stress has been proposed to cause renal disease in captive, compared to free-ranging, wild cats^40^. Renal disease is an age-related disease in all felids and other species studied to date^40–43^. It would be reasonable to conclude that the average age of the Scottish Wildcats in our sample, despite being adult, was less than the average age of our domestic cats, limiting the opportunity for renal lesions to develop. Likewise, in our study, cats and dogs with CIN were generally older than those without CIN. Nevertheless, the size of the whole wildcat kidneys (4–6cms long) obtained in our study would suggest that these animals were at least full-grown adults.

### Kidney trace element profile does not suggest a causal link to CKD

#### Arsenic

Previous work by us had determined higher levels of total arsenic being measured in commercially available, complete pet foods containing fish^30^, which are more commonly fed to pet cats than to pet dogs. The evolutionary history of the domestic cat, originating from the Near Eastern desert region^37^, suggests that fish were never a common food source. Thus, the domestic cat may not be physiologically adapted to constituents found at high levels in fish, such as arsenic, mercury^44^ and iodine^45,46^. In the present study, we were unable to determine diet histories and thus were unable to associate fish intake with kidney tissue arsenic content. The higher levels of total arsenic in kidney tissue and urine in domestic cats compared to dogs and Scottish wildcats, likely reflect dietary intake over the previous few months and days, respectively prior to either the planned euthanasia or death (in case of wildcats). In addition, domestic cats and dogs could have reduced their intake in the days prior to euthanasia, which could influence (reduce) our urine, but not kidney, elemental levels. Nevertheless, the Scottish wildcat is unlikely to have ever consumed a commercial diet, nor any wild fish; instead obtaining its food by hunting wild rabbits, rodents and birds^36^, which may explain its lower renal accumulation of arsenic compared to the domestic cat.

The kidney excretes the majority of any absorbed arsenic in the urine within 24–48 hours of its consumption^26,47,48^, thus spot samples of urine collected at necropsy are likely to reflect arsenic intake over the few days prior to death, rather than being indicative of chronic intake. Greater urinary arsenic excretion has, however, been associated with increased dietary intake^48^ and an increased risk of renal dysfunction and CKD^27^. Indeed, in the current study, urinary arsenic concentrations were significantly greater in cats and dogs with CIN compared to those without CIN, suggesting an association with a risk factor for CKD in the companion animal population. However, we found no consistent stratified increase in kidney tissue arsenic content and stage of CIN. Kidney levels of arsenic were, in general, very low. However, while urinary arsenic concentrations have been measured in other species and has been associated with renal disease; to our knowledge, the kidney arsenic content was unknown. Therefore, conclusions on any relationship between increased intake of fish-based foods, longer-term arsenic ingestion and renal bioaccumulation with the development of CKD in the domestic cat population are not reliably supported by the current study. Arsenic accumulates primarily in the kidney but also in other organs (liver > pancreas > aorta > testis)^20^. Thus, our data do not exclude the possibility that renal tissue arsenic content biomarks ectopic accumulation. High arsenic intake, particularly of inorganic forms, is known to cause kidney disease and bladder cancer, often underpinned by extensive oxidative damage^49–51^.

#### Arsenic in fish-containing pet foods is primarily an organic, non-toxic form

High total arsenic concentration in fish-containing wet foods for cats, as determined by us previously^30^, is almost entirely organic AsB. Only minute amounts of DMA and MMA were observed. As(III) and As(V), both of which are highly toxic, were not-detectable in any food, similar to previously published studies on arsenic in fish for human consumption^33^. Thus, whilst it is acknowledged that some fish-based pet foods have higher than average levels of arsenic content, despite most being within legal limits, the type of arsenic species are predominantly organic forms that are unlikely to represent any biological risk to companion animals.

#### Iron

iron is a known catalyst in the formation of ROS^52^ and is therefore implicated in the development of oxidative stress which can promote renal injury^53,54^. Iron is not normally stored in the kidneys in great quantities and is only minimally excreted in urine since most circulating iron is bound to proteins such as the iron-transport protein, transferrin, and iron that does pass into the renal tubules is mostly reabsorbed^55^. Increased levels of iron have been found in the kidney tissue, associated with glomerular lesions^56,57^, and urine^53,58^ of human CKD patients compared to humans without renal disease. However, we measured lower concentrations of iron in cat, compared to dog, kidneys and, in both species, in kidneys with CIN compared to those without. Furthermore, we saw no difference in urinary iron concentrations between species or between healthy animals and those with CIN in our study.

### Anti-oxidant minerals are low in cat versus dog kidneys

#### Copper and zinc

While elevated levels of pro-oxidant minerals increase the likelihood of oxidative damage, this phenomenon occurs due to an imbalance between these and the anti-oxidant defences that are in place within the tissues. Together, copper and zinc comprise the functional core of superoxide dismutase (SOD) isoforms (SOD1 and SOD3) – a potent antioxidant enzyme^11^. SOD1 is active in the cytosol while SOD3 activity occurs extracellularly^59^. The former constitutes 80% of the total SOD activity in the mammalian kidney^60^, thus the importance of copper and zinc is clear. Yet, in the present study, these minerals were both depleted in cat versus dog kidneys and copper was also decreased in kidneys with CIN compared to healthy kidneys. It is possible therefore that felids may have lower levels of SOD1 and SOD3 in their kidney *per se*, which could predispose them to oxidative damage which, over time, progresses toward chronic renal disease. Furthermore, a recent study showed that cats with CKD had lower levels of zinc stored in their kidneys compared to cats without renal disease^61^. However, the zinc content of kidney tissue in the current study did not differ with the presence or absence of CIN. Nonetheless, the lower levels of zinc stored in the cats’ kidneys, *per se*, may contribute to the cat’s propensity to develop CKD more readily than dogs.

Zinc is excreted primarily in faeces and only very small amounts are excreted into the urine by the kidney^62^. Thus, despite measuring greater zinc concentrations in dog, versus cat, urine, this may not reflect dietary concentrations of the mineral directly. However, in cats and dogs^62^ and rats^63^, kidney zinc levels have been shown to increase with increased dietary intake of this trace element. Another implication of our findings might therefore be that cats receive less zinc from their diet than dogs. Dietary histories were not obtained for the animals in our study and our previous work showed no significant difference in the zinc content of commercially-available, complete foods for cats and dogs^30^. Nonetheless, relatively lower zinc intake could lead to increased oxidative stress in tissues, while adequate and increased dietary zinc could have a protective effect, as has been demonstrated in kidneys of diabetic mice^64^. However, excess dietary zinc intake has been shown to reduce renal function, induce systemic hypertension and reduce blood flow^65^. Importantly, pet foods appear to comply with maximum and minimum recommended levels of zinc^30^.

### Multi-elemental analysis reveals further mineral differences in domestic cat vs. dog vs. Scottish wildcat kidneys

In the current study, magnesium, calcium and phosphorus, strontium and rubidium were each significantly different between groups. Lower magnesium content of cat kidneys (cf. dogs) is of interest: reduced intake of magnesium has been associated with reduced renal function^66^, increased risk of CKD in cats^67^ and increased fibroblast growth factor 23 (FGF23)^68^. Higher FGF23 can induce renal interstitial fibrosis^69^. Low serum magnesium is associated with an increased risk of death in cats^70^. Equally, nephrocalcinosis – deposits of calcium and phosphate in the kidneys – can be both a consequence and cause of renal disease^71^. We found cat and dog kidneys with CIN had higher calcium content, but in no sample was deposition to an extent that crystals were evident. No *a priori* hypotheses in regard to the biological role or toxicity of rubidium and strontium were proposed. Rubidium tends to follow potassium levels in the kidney and, generally, higher levels are reno-protective^72^. We found urinary rubidium and strontium levels to trend higher in dogs, a species less prone to renal disease, than cats. Lower levels of these two elements in cat, compared to dog, kidneys may imply that the feline kidney is less protected, although this would require further validation in other studies. Nevertheless, it is notable that tissue Rb in Scottish wildcats was double that of domestic cats, perhaps indicative of higher potassium intake and greater reno-protection.

In conclusion, domesticated cats, but not wildcats, have a high incidence of CIN, as compared to domesticated dogs. Furthermore, domesticated cats, but not wildcats or domesticated dogs, tend to be fed more fish-based foods, which have higher arsenic content, although this is primarily of a non-toxic form. Whilst domesticated cats, compared to dogs, do have increased renal and urinary levels of arsenic, indicative of greater dietary intake, the absolute quantities are very low, do not stratify with severity of CIN and are therefore unlikely to underpin the high incidence of CKD in this species. Nevertheless, our complete mineral profiling of cat and dog kidneys does reveal significantly lower concentrations of antioxidant minerals, such as copper and zinc, or potentially renal-protective minerals, such as rubidium and strontium, in domesticated cats relative to dogs. CKD is an age-related disease and an extended exposure to a pro-oxidant renal environment, due to diet or genetics, may be contributory toward renal disease in the domestic cat.

## Supplementary information

Supplementary Information.
