# Supplementary material for: Renal accumulation of prooxidant mineral elements and CKD in domestic cats
Source: Sci Rep. 2020 Feb 21;10:3160. doi: 10.1038/s41598-020-59876-6 (PMC7035273; doi:10.1038/s41598-020-59876-6)
Supplement: Supplementary file 1 — Supplementary Information. [file 41598_2020_59876_MOESM1_ESM.pdf]

Arsenic content of kidneys and urine from cats and dogs with or without chronic interstitial nephritis

Authors: Alborough, R.[1*], Grau-Roma, L.[1,3], De Brot, S.[1,3], Hantke, G.[2], Graham, P.[1] and Gardner D.S.[1*]

Supplementary Information

Figure S1: Representative images of CIN and renal fibrosis

Figure S2. Chromatograms of arsenic species standards.

Figure S1: Representative images of chronic interstitial nephritis (CIN) (left, haematoxylin eosin stain) and renal fibrosis (right, Masson's trichrome stain)

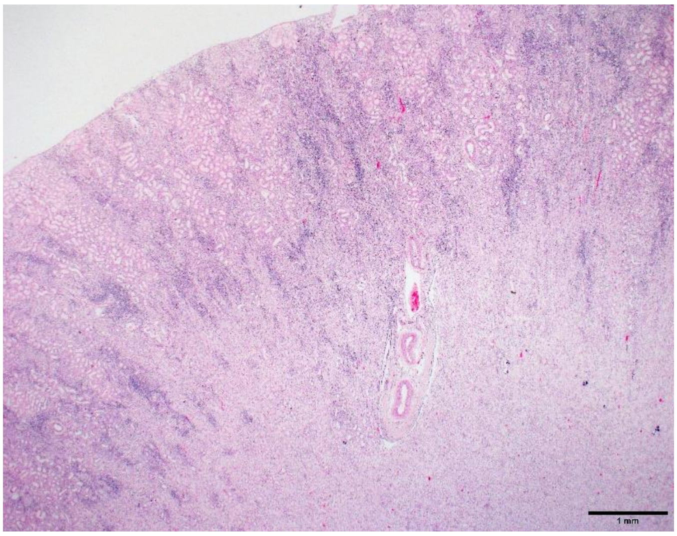

Figure 1a. Severe CIN.

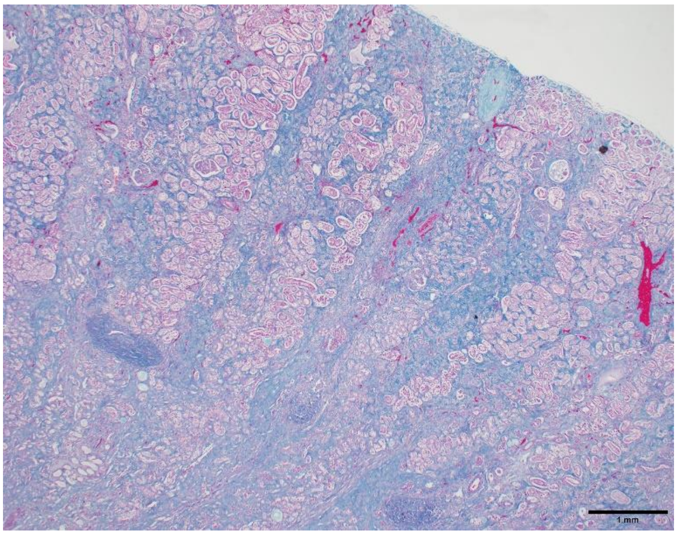

Figure 1b. Severe interstitial fibrosis

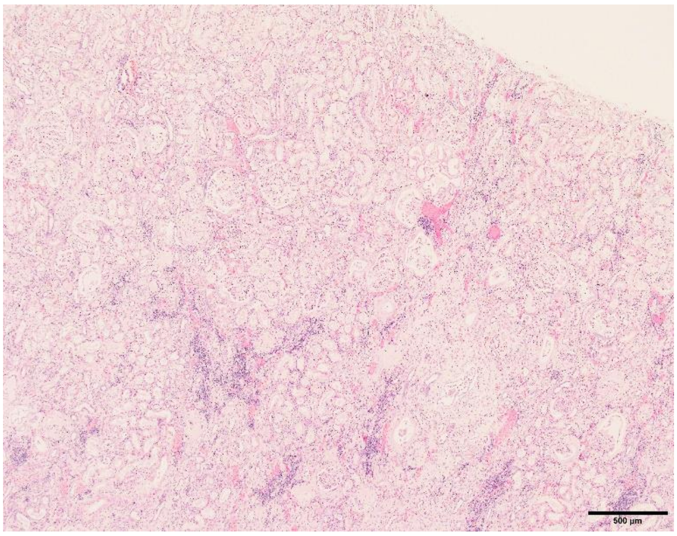

Figure 1c. Moderate CIN

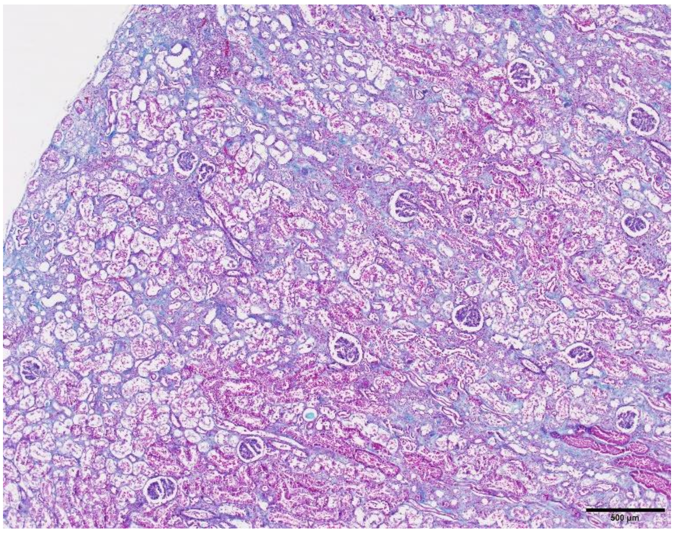

Figure 1d. Moderate interstitial fibrosis

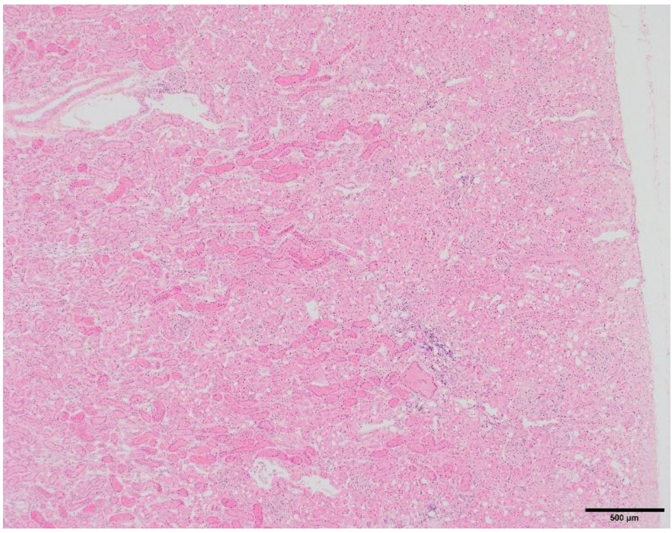

Figure 1e. Mild CIN

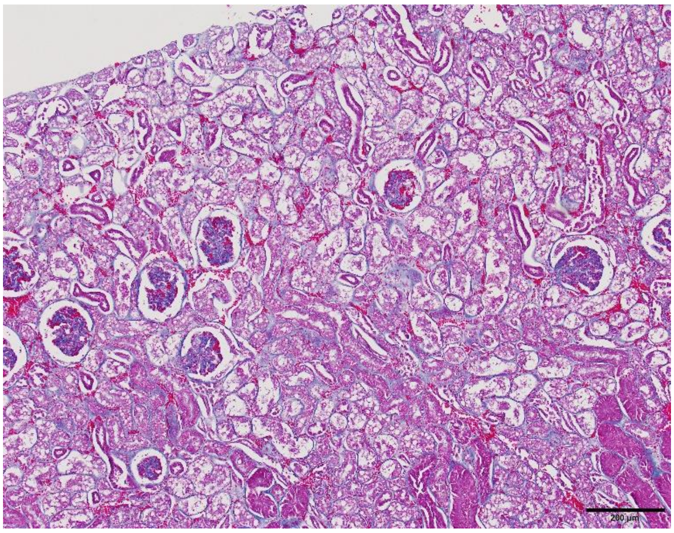

Figure 1f. Mild interstitial fibrosis

Figure S1 legends

Figure S1a. Severe CIN. There is abundant, multifocal to coalescing infiltration of lymphocytes and plasma cells within the interstitium of mostly the renal cortex, affecting more than 25% of the tissue section. Hematoxylin and eosin.

Figure S1b. Severe interstitial fibrosis. There is abundant and diffuse deposition of collagen (blue stain) within the renal interstitium associated with marked loss and atrophy of renal tubules. Masson's trichrome stain.

Figure S1c. Moderate CIN. There are low to moderate numbers of lymphocytes and plasma cells multifocally distributed within the renal interstitium, affecting between 5 and 25% of the tissue section. Hematoxylin and eosin.

Figure S1d. Moderate interstitial fibrosis. There is multifocal to coalescing deposition of moderate amount of collagen (blue stain) within the renal interstitium associated with loss and atrophy of renal tubules. Masson's trichrome stain.

Figure S1e. Mild CIN. Low numbers of lymphocytes and plasma cells are multifocally present within the renal interstitium, affecting less than 5% of the tissue section. Hematoxylin and eosin.

Figure S1f. Mild interstitial fibrosis. There is multifocal deposition of low amount of collagen (blue stain) within the renal interstitium. Masson's trichrome stain.

Figure S2. Chromatograms of arsenic species standards.

(a) Standard 1

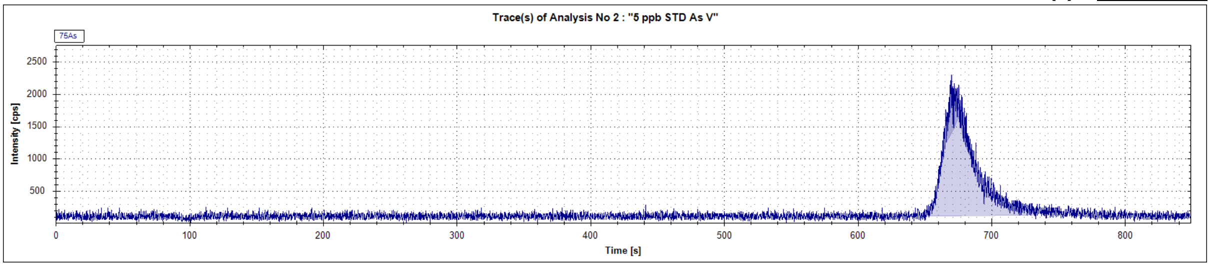

(b) Standard 2

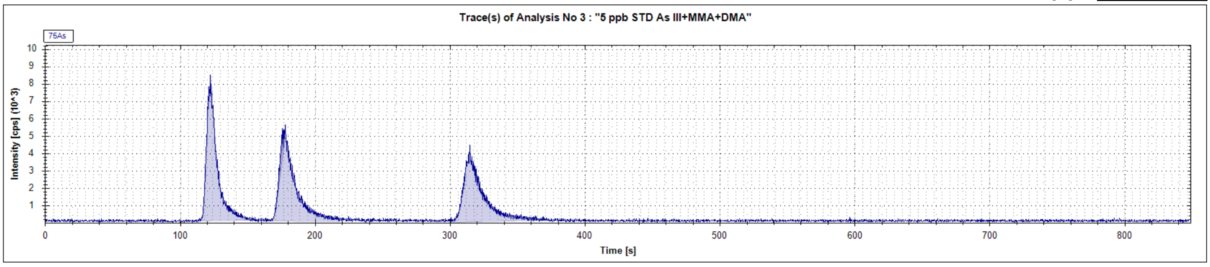

(c) Standard 3

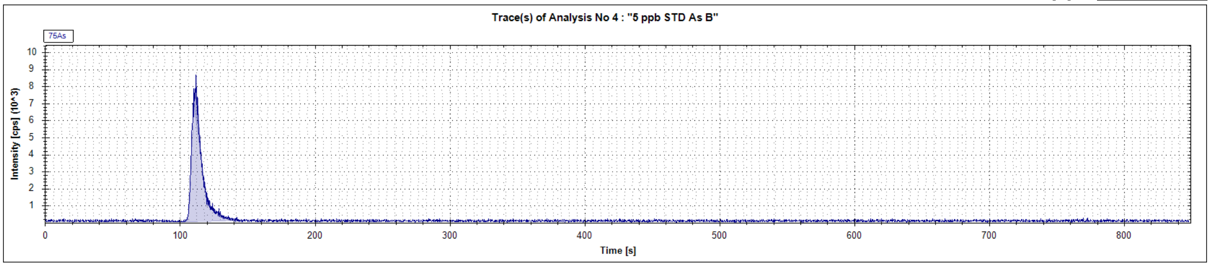

(d) Standard 4

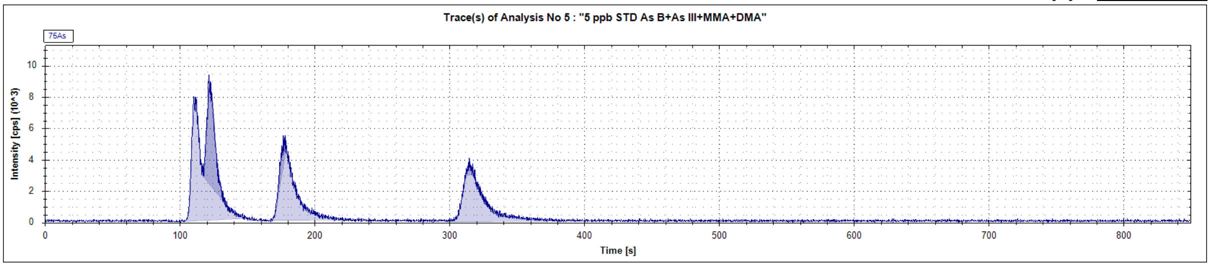

FigureS2: Chromatograms of standard solutions of **(a)** 5.0 µg/L arsenate (As V), **(b)** 5.0 µg/L arsenite, monomethylarsinic acid and dimethylarsinic acid (As III + MMA + DMA), **(c)** 5.0 µg/L arsenobetaine (As B), and **(d)** 5.0 µg/L arsenobetaine, arsenite, monomethylarsinic acid and dimethylarsinic acid (AsB + As III + MMA + DMA) Peaks indicating the presence of arsenobetaine, arsenite, dimethylarsinic acid, monomethylarsinic acid and arsenate appeared at retention times ~ 110 s, 125 s, 175 s, 310 s and 660 s, respectively.
